# Detection of De Novo Dividing Stem Cells In Situ through Double Nucleotide Analogue Labeling

**DOI:** 10.3390/cells11244001

**Published:** 2022-12-10

**Authors:** Sheed Itaman, Grigori Enikolopov, Oleg V. Podgorny

**Affiliations:** 1 Center for Developmental Genetics, Stony Brook University, Stony Brook, NY 11794, USA; 2 Graduate Program in Neurobiology, Stony Brook University, Stony Brook, NY 11794, USA; 3 Department of Anesthesiology, Stony Brook School of Medicine, Stony Brook, NY 11794, USA; 4 Shemyakin-Ovchinnikov Institute of Bioorganic Chemistry, Russian Academy of Sciences, 117997 Moscow, Russia; 5 Center for Precision Genome Editing and Genetic Technologies for Biomedicine, Pirogov Russian National Research Medical University, 117997 Moscow, Russia; 6 Koltzov Institute of Developmental Biology, Russian Academy of Sciences, 119334 Moscow, Russia; 7 Federal Center of Brain Research and Neurotechnologies, Federal Medical Biological Agency, 117997 Moscow, Russia

**Keywords:** stem cell quiescence, proliferation, thymidine analogues, adult hippocampal neurogenesis, radial glia-like stem cells, memantine

## Abstract

Tissue-specific somatic stem cells are characterized by their ability to reside in a state of prolonged reversible cell cycle arrest, referred to as quiescence. Maintenance of a balance between cell quiescence and division is critical for tissue homeostasis at the cellular level and is dynamically regulated by numerous extrinsic and intrinsic factors. Analysis of the activation of quiescent stem cells has been challenging because of a lack of methods for direct detection of de novo dividing cells. Here, we present and experimentally verify a novel method based on double labeling with thymidine analogues to detect de novo dividing stem cells in situ. In a proof of concept for the method, we show that memantine, a drug widely used for Alzheimer’s disease therapy and a known strong inducer of adult hippocampal neurogenesis, increases the recruitment into the division cycle of quiescent radial glia-like stem cells—primary precursors of the adult-born neurons in the hippocampus. Our method could be applied to assess the effects of aging, pathology, or drug treatments on the quiescent stem cells in stem cell compartments in developing and adult tissues.

## 1. Introduction

Quiescence, a cellular state of reversible cell cycle arrest, is a hallmark of many types of somatic stem cells in adult organisms that distinguishes them from actively proliferating intermediate progenitors, senescent cells, or terminally differentiated cells in irreversible cell cycle arrest. Quiescent stem cells periodically enter the cell cycle and consequently support tissue renewal or repair. This mode of proliferative behavior has been observed in hematopoietic, neural, muscle, hair follicle, intestinal and other types of tissue-specific stem cells, as well as transformed cells that support tumor growth [1,2,3,4,5].

The quiescence of somatic stem cells may help preserve the integrity of their genomes by preventing the accumulation of potentially deleterious mutations arising from active DNA replication [4]. The quiescent state of stem cells, as well as their entry into the cell division cycle, is actively supported by a range of cell-autonomous and extrinsic signals. The balance between the quiescence and proliferation of stem cells ultimately determines the rate of tissue renewal and repair, and the maintenance of the stem cell pool throughout the lifespan. Aging, disease, or therapy may disturb this balance, thus causing premature depletion of the stem cell pool and a loss of tissue homeostasis [6,7,8]. The importance of stem cells in health and disease underscores the need for revealing the basic framework of stem cell quiescence and division.

The infrequency of stem cell division can be used for identifying and studying these cells in situ, according to the basic premise that cells should incorporate and then retain division-dependent tags such as modified nucleotides or fluorescent proteins. Such tags will be gradually diluted by multiple cycles of cell division and hence retained only in cells that divide rarely, e.g., stem cells. One approach for identifying stem cells in situ is based on cumulative labeling of duplicating DNA with a modified nucleotide, usually a thymidine analogue, followed by a prolonged chase period during which the DNA label is diluted to undetectable levels in actively dividing cells but retained in the infrequently dividing stem cells (label-retaining cells, or LRCs) [9,10,11,12]. An important limitation of this approach is that it is retrospective: it can demonstrate that a particular LRC was in S phase at least once during the period of the cumulative labeling and underwent only few cycles of division during the chase period, but it cannot report on the rate of recruitment of quiescent stem cells into the cell cycle. Another approach for identifying and following stem cells relies on a fusion between histone H2B and green fluorescent protein (H2B-GFP), expressed under the control of a tetracycline-responsive regulatory element [12,13,14,15]. Treatment with doxycycline elicits transient expression and nuclear accumulation of H2B-GFP, whose fluorescence decays during a chase period to undetectable levels in actively proliferating cells but persists in quiescent or slowly dividing cells. This approach can be combined with use of a proliferation marker (a modified nucleotide or the Ki67 protein) to determine the shifts in the balance between quiescence and proliferation of stem cells [13]. Both LRC assays, nucleotide-based or GFP-based, are retrospective and rely on protracted chase periods to achieve a state in which only slowly dividing or quiescent stem cells remain labeled while their progeny loses the labeling tag.

Here, we present a novel method for detecting de novo dividing stem cells in situ. This method is based on a combination of cumulative and pulse-chase tagging of cells with two thymidine analogues and detects stem cells that have exited the quiescent state and entered the cell cycle de novo. To provide a proof of concept, we validated this method in a model of neural stem cells in the hippocampal dentate gyrus (DG) of the adult mouse brain and demonstrated its application in revealing cellular mechanisms underlying the enhancement of adult hippocampal neurogenesis (AHN) by the Alzheimer’s disease drug memantine. This approach could be universally applied to any type of developing, adult, or neoplastic tissue.

## 2. Materials and Methods

### 2.1. Animals

Experiments were performed in 2-month-old heterozygous Nestin-GFP male mice [16]. Mice were maintained in groups (three or four mice per cage) in an animal facility approved by the Association for the Assessment and Accreditation of Laboratory Animal Care International (AAALAC) at Stony Brook University. All procedures with animals were approved by the Institutional Animal Care and Use Committee (approval no. 954192). Mice were housed under an ambient temperature of 22–24 °C with a 12 h light/dark cycle and were given ad libitum access to food and water. All experimental procedures were approved by the Stony Brook University Animal Use Committee and performed according to the NIH guidelines.

### 2.2. Delivery of Nucleotide Analogues

For the cumulative labeling of dividing cells, mini osmotic pumps (Alzet, California, USA, model 1007D, volume ~100 µL, pump performance ~0.5 µL/h) filled with 5-bromo-2′-deoxyuridine (BrdU) (Sigma-Aldrich, St. Louis, MO, USA, #B5002) were implanted subcutaneously in the midback region of each animal. Mini osmotic pumps were filled under aseptic conditions with BrdU dissolved in sterile dimethyl sulfoxide (DMSO) (Sigma-Aldrich, St. Louis, MO, USA, #D2650) according to the manufacturer’s recommendations. Appropriate filling was verified by weighing the pumps before and after the pump charging, taking into account the density of DMSO. Before implantation, pumps were “primed” by incubation in a saline solution for at least 6 h at 37 °C. Mice were anesthetized with isoflurane (3% for induction and 1.5% for maintenance). The midback region of each animal was shaved and decontaminated with 70% ethanol. The skin was incised with a length of approximately 1 cm. Subsequently, space for a pump was created under the skin using forceps. A pump was placed into the formed space, and the wound was closed with 7 mm Reflex metallic clips (Alzet) with a clip applier and treated with iodine wound spray. After surgery, the mice were returned to their home cages. For analgesia, mice received three injections of buprenorphine dissolved in sterile saline at a dose of 0.1 mg/kg: one injection occurred immediately after surgery, and two subsequent injections were administered 24 h apart. The implanted pumps continuously delivered BrdU at a rate of 50 mg/kg per day for 7 days. To interrupt delivery of BrdU at the desired timepoints, we removed the implanted pumps. Mice were anesthetized with isoflurane (3% for induction and 1.5% for maintenance). The wound was decontaminated with 70% ethanol, metallic clips were gently removed with a clip remover, and the wound was reopened. The pump was extruded, and the wound was closed with clips and treated with iodine wound spray. Mice received an injection of buprenorphine immediately after surgery and were returned to their home cages. For pulse labeling of dividing cells, the second label, a thymidine analogue 5-ethynyl-2′-deoxyuridine (EdU) (Thermo Fisher Scientific, Waltham, MA, USA, #A10044) dissolved in sterile saline was intraperitoneally injected at a dose of 123 mg/kg (6.15 mg/mL). The dose of EdU was equimolar to the saturating dose of 150 mg/kg BrdU [17].

The rationale for the delivery of BrdU via mini osmotic pumps is that it provides several advantages over the common delivery methods, such as intraperitoneal injections of BrdU or treatment with BrdU dissolved in drinking water. Repeated intraperitoneal injections should be performed at least three times per day for seven days to label all dividing cells [18] and can be highly stressful for the mice and thereby may affect cell proliferation in the SGZ [19]. In contrast, two very rapid (1 min each) subcutaneous surgeries (implantation and removal of a mini osmotic pump) under inhalation anesthesia appear to be less stressful than repeated daily intraperitoneal injections. Delivery of BrdU via drinking water was found to label different numbers of dividing cells during the light and dark phases of the day because of the circadian dependence of water intake, and, critically, to label only 7–15% of the SGZ cells that are in the S phase; moreover, the intensity of the signal in BrdU-positive nuclei was significantly lower than in animals which received injections of BrdU [20]. Thus, there is a significant risk that a large fraction of dividing cells will remain unlabeled if the label is administered perorally. Therefore, using the mini osmotic pumps, with a constant rate of BrdU delivery at low doses, helps to circumvent the potential caveats of other methods of long-term administration of the modified nucleotides to the animals.

### 2.3. Memantine Injections

Memantine hydrochloride (Sigma-Aldrich, St. Louis, MO, USA, #M9292) dissolved in a sterile saline was injected intraperitoneally. Each single intraperitoneal injection delivered memantine at a dose of 50 mg/kg (2.5 mg/mL).

### 2.4. Brain Sample Collection and Tissue Processing

To collect brains for subsequent microscopic analysis, we deeply anesthetized mice through intraperitoneal injection of a mixture of ketamine and xylazine dissolved in sterile saline at doses of 100 and 20 mg/kg, respectively. Anesthetized mice underwent transcardiac perfusion with 0.01 M phosphate-buffered saline (PBS, pH 7.4) followed by cold 4% paraformaldehyde in PBS (pH 7.4) according to standard procedures. Brains were extracted from skulls and postfixed overnight at 4 °C. Next day, the brains were washed with PBS, and the right hemispheres were sliced sagittally in a lateral-to-medial direction with a Leica VT1200S vibratome (Leica-Microsystems, Wetzlar, Germany). Slices (thickness of 50 µm) were consecutively collected into six wells of a 24 well plate filled with PBS according to the fractionator principle, as previously described [21,22]. Thus, each well contained a series of slices (9–11 slices) representing a fraction (one sixth) of the entire hippocampal DG of one hemisphere.

### 2.5. Visualization of Labels and Cell Type Specific Markers in Series of Brain Slices

To detect BrdU and EdU in mouse brains, we used our protocol for triple S phase labeling [23]. For each animal, a series of free-floating slices (one well of a 24 well plate) was transferred into a well of a new 24 well plate. The incubation volumes for antibody and click reaction mixtures were 500 µL. All staining procedures, excluding DNA denaturation in hydrochloric acid solution, were conducted with gentle shaking at room temperature in the dark, and three washes with PBS were performed between steps. Slices were initially permeabilized in 4% Triton X-100 (Sigma-Aldrich, St. Louis, MO, USA, #T8787) in PBS for 1 h. They were initially incubated for 15 min in the first click reaction mixture (20 mM (+)-sodium L-ascorbate (Sigma-Aldrich, St. Louis, MO, USA, #A4034), 10 µM Alexa 555-azide (Thermo Fisher Scientific, Waltham, MA, USA, #A20012) and 4 mM copper sulfate in PBS) to detect EdU with a fluorescent azide and subsequently in the second click reaction mixture (20 mM (+)-sodium L-ascorbate, 2 mM azidomethyl phenyl sulfide (Sigma-Aldrich, MO, USA, #244546) and 4 mM copper sulfate in PBS) to block non-specific binding of primary antibodies against halogenated thymidine analogues. Subsequently, slices were exposed to 2 N hydrochloric acid for 40 min at 37 °C for DNA denaturation, then incubated in 0.1 M borate (pH 8.0) twice for 10 min for neutralization of the acid (no washing in PBS). Slices were blocked in 5% goat serum (Sigma-Aldrich, St. Louis, MO, USA, #G9023) in PBS containing 0.2% Triton X-100 for 1 h. The same solution was used for preparing the antibody mixtures. Slices were incubated with an antibody mixture containing mouse monoclonal anti-BrdU antibody clone B44 (1:1000) (BD Biosciences, NJ, USA #347580), chicken anti-GFP antibody (1:400) (Aves Labs, CA, USA, #GFP-1020) and rabbit polyclonal anti-GFAP antibody (1:400) (DAKO, #Z0334) overnight. The next day, slices were stained with a secondary antibody mixture containing goat anti-mouse IgG (H + L) antibody conjugated with DyLight™ 405 (1:400) (Jackson ImmunoResearch, Cambridgeshire, UK, #115-475-166), goat anti-chicken IgY (H + L) antibody conjugated with Alexa Fluor 488 (1:500) (Thermo Fisher Scientific, Waltham, MA, USA, #A11039) and goat anti-rabbit IgG (H + L) antibody conjugated with Alexa Fluor 647 (1:1200) (Thermo Fisher Scientific, Waltham, MA, USA, #A 21236) for 2 h. Stained brain slices were attached to gelatin-coated glass-slides, mounted in Fluorescence Mounting Medium (DAKO, Glostrup, Denmark, #S3023) and covered with coverslips.

### 2.6. Confocal Microscopy

Preparations of brain slices were imaged with a PerkinElmer spinning disk confocal microscope equipped with laser lines at 405, 488, 561 and 640 nm, and a 40× Nikon objective with a numerical aperture of 0.95. Four-channel confocal images were acquired with appropriate barrier filters in Volocity software (Improvision Ltd., Coventry, UK). On each slice, we identified the DG and assigned upper and lower limits for capturing a Z-stack. For all preparations, the step size of the Z-stack was set to 1 µm. Staining procedures elicited shrinking of brain slices. Therefore, Z-stacks typically contained 28–32 optical sections. Z-stacks acquired with 10% overlap in the XY-plane were stitched in Volocity software to obtain large images of the whole DG in a given slice.

### 2.7. Cell Counting

Cells in the subgranular zone (SGZ) of the DGs that incorporated labels were identified in confocal images and manually counted. Cell counting was performed by blinded investigators. On each confocal image of the DG, we identified the SGZ between the granule cell layer with the Nestin-GFP channel. By scrolling XY-planes along the Z-stack and switching between individual channels or their combinations, we identified, determined the phenotypes of, and counted cells that incorporated labels. We considered labeled cells within the uppermost focal plane as boundary-touching objects and therefore excluded them from counting. We counted labeled cells throughout the SGZ identified in a particular confocal image. Cell counts obtained for all confocal images of a given experimental series were summed and then extrapolated to the entire hippocampal DG of both hemispheres through multiplication by 12 (six wells per hemisphere and two hemispheres per brain).

### 2.8. Data Analysis

All quantitative data are expressed as mean ± standard error of mean (SEM) and were subjected to statistical analysis. We used unpaired Mann–Whitney’s non-parametric test for the determination of significant differences in cell numbers between the control and experimental groups. Differences between the means of experimental groups with *p*-values below 0.05 were considered significant.

## 3. Results

### 3.1. A New Labeling Scheme for Visualizing De Novo Dividing Cells

**Figure 1:**
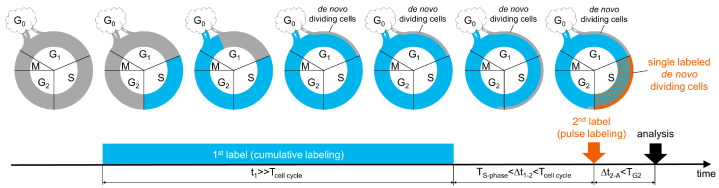
Labeling scheme for the unequivocal detection of de novo dividing stem cells. G_1_, S, G_2_ and M: phases of the cell cycle. G_0_: a state outside the cell cycle. t_1_: duration of cumulative labeling with the first nucleotide analogue. ∆t_1–2:_ a time window between the termination of cumulative labeling with the first nucleotide analogue and pulse labeling with the second nucleotide analogue. ∆t_1-A_: a time window between pulse labeling with the second nucleotide analogue and the collection of samples for analysis. Details are in the main text.

In a hypothetical stem cell cascade, quiescent stem cells periodically enter the cell cycle, thereby renewing their own population or supporting the population of actively proliferating intermediate progenitors (Figure 1). After completion of one or more cycles of division, renewed stem cells return to the quiescent state, while the intermediate progenitors (with or without additional cell divisions) irreversibly leave the cell cycle and differentiate and mature into specific cell phenotypes. Our proposed labeling scheme (Figure 1) is based on the assumption that stem cells reside in the quiescent state considerably longer than a single cell cycle. If the first nucleotide analogue is continuously delivered, cells that have passed through S phase of the cell cycle at least once become tagged with that nucleotide. After mitosis, the tagged stem cells either leave the cell cycle or reenter G_1_ phase and continue division. Simultaneously, a cohort of untagged quiescent cells enters the G_1_ phase of the cell cycle. These de novo dividing cells progress through the cell cycle, reaching S phase, in which they incorporate the nucleotide analogue and also become tagged. Because the population of proliferating cells may have non-homogeneous cell cycle lengths, to avoid detection of cells with prolonged cell cycles, we performed cumulative labeling with the first nucleotide analogue for much longer than the average cell cycle duration (T_cell cycle_) of the proliferating population (Figure 1). Thus, continuous cumulative labeling marks almost all cells in the cell cycle except for a cohort of de novo dividing cells in their first G_1_ phase after leaving the quiescent state. After instant termination of the first nucleotide analogue delivery, this cohort of untagged de novo dividing cells enters S phase. Pulse delivery of the second nucleotide analogue after a time interval (∆t_1–2_) longer than the S phase duration (T_S-phase_) but shorter than the cell cycle length (T_cell cycle_) marks all cells in S phase at that point. Consequently, the cells that underwent at least one division during the cumulative labeling are tagged by both labels while the cohort of de novo dividing cells is tagged with only the second label (Figure 1). The time window (∆t_1–2_) between the termination of cumulative labeling and the pulse labeling ensures that the cohort of untagged de novo dividing cells passed their first S phase but have not reached their second S phase after cell cycle reentry. Samples are collected for analysis before the cohort of single labeled de novo dividing cells reaches mitosis (∆t_2-A_). Next, the phenotypes of cells labeled with only the second tag are determined in situ, and cells are quantified.

### 3.2. Detection of De Novo Dividing Stem Cells in the Hippocampal DG

**Figure 2:**
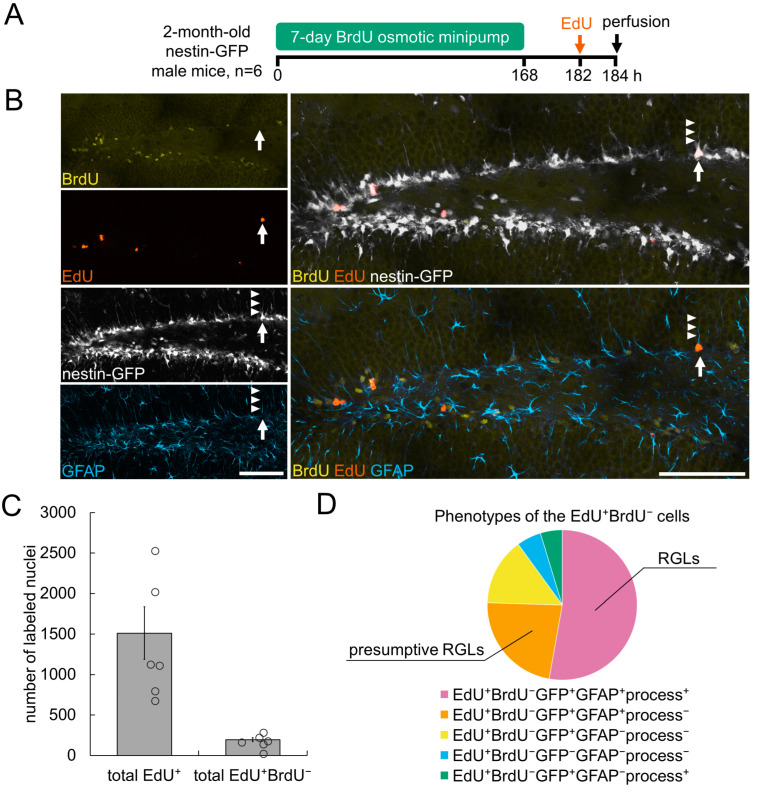
Validation of the double labeling method for the detection of de novo dividing RGL stem cells in the hippocampal dentate gyrus in adult mice. (**A**) Scheme of double labeling for the detection of de novo dividing cells. (**B**) Representative image of the DG sample with a de novo dividing RGL stem cell (EdU^+^BrdU^−^ with the phenotype GFP^+^GFAP^+^process^+^) in its first S phase (arrow) (see also Figure S1). The image is an optical section captured with a confocal microscope. Arrowheads indicate an apical process of the de novo dividing RGL stem cell. Scale bars: 100 µm. (**C**) Total number of cells in S phase and the number of de novo dividing cells (cells that had incorporated EdU but not BrdU). Data are presented as mean ± SEM. (**D**) Phenotyping of de novo dividing cells indicated that most were RGL stem cells (Figure S2 and Table S1).

We next sought to validate our double-labeling scheme by applying it to hippocampal neurogenesis in the adult mouse brain, a well characterized model of stem cell maintenance and division. We relied on the reporter Nestin-GFP transgenic mouse line, which has been thoroughly characterized [16,24,25,26] and used for the identification and investigation of a wide range of tissue-specific stem cells [27,28,29,30,31,32,33]. We applied the scheme in Figure 1 to 2-month-old Nestin-GFP male mice (n = 6) with continuous delivery of BrdU as the first tag and pulse injection of EdU as the second tag, followed by identification, phenotyping and counting of cells with EdU^+^BrdU^−^ nuclei in the SGZ on confocal images of the hippocampal SGZ (Figure 2A).

We continuously delivered BrdU for 7 days via mini osmotic pumps implanted subcutaneously (Figure 2A). Continuous delivery of BrdU marked all dividing cells that passed S phase at least once during the 7-day period. Reported estimates of the cell-cycle length of progenitor cells within the neurogenic niche in the hippocampal DG range from 20 to 30 h [34,35,36]. Therefore, a 7-day period corresponds to approximately 6–8 cell division cycles. Previous studies have estimated that quiescent radial glia cells (RGLs) in the hippocampal DG, after their activation, undergo on average of two to four consecutive asymmetric divisions to generate transit amplifying neural progenitors (ANPs), which in turn undergo an average of two or three additional rounds of symmetric divisions [23,36,37,38].

Hence, a 7-day exposure to BrdU encompasses the duration of at least one round of newborn cell generation from a quiescent stem cell, and cells at different stages of the proliferation cascade are marked with BrdU. Delivery of BrdU and therefore tagging the cells with this modified nucleotide was terminated by the removal of the mini osmotic pump [19]. Next, the mice received a single pulse intraperitoneal delivery of the second label (EdU) 14 h after pump removal, and mouse brains were collected for analysis 2 h later (Figure 2A). Thus, all cells in S phase when the second label was delivered were tagged with EdU. In this double labeling scheme, most cells that incorporated EdU were co-labeled with BrdU because these cells had undergone S phase at least once and at least one round of division during the preceding 7 days. However, a smaller cohort of cells that incorporated EdU were expected to be free of the BrdU label. The cells of this cohort, after a period of quiescence, entered the cell cycle within the 14 h time window when neither the first nor the second label was available for incorporation, and were in S phase at the time of EdU administration. The rationale for choosing the 14 h time window between the completion of the first label delivery and the second label injection was that it encompasses the S phase duration of hippocampal neural progenitors, which has been estimated to be 10–12 h [23,34,35,36]. Hence, the 14 h time window enabled cells unlabeled with the first label, BrdU, to enter and complete S phase, and become marked when the second label, EdU, was delivered.

Therefore, all cells marked with the second label (EdU) but not the first label (BrdU) were not in the cell cycle within the prior 7 days and, when the brains were collected, were in their first S phase, i.e., such cells were dividing de novo after a period of quiescence.

The EdU^+^ nuclei contained a subset of nuclei that were not labeled with BrdU (Figure 2B and Figure S1). These EdU^+^BrdU^−^ nuclei represented ~12% of the total number of EdU^+^ nuclei (Figure 2C) (194 ± 25 of EdU^+^BrdU^−^ nuclei among 1512 ± 325 of EdU^+^ nuclei). We performed phenotyping of the identified cells in the SGZ with EdU^+^BrdU^−^ nuclei by assessing the following features characteristic of RGLs [16,21,36,39,40,41]: (i) signal of the Nestin-GFP transgene in and around nuclei (scored as GFP^+^ or GFP^−^), (ii) the presence of an apical GFP-positive process reaching at least the middle of the granule cell layer in a given field of view (scored as process^+^ or process^−^), and (iii) a signal of an astrocytic marker glial fibrillary acid protein (GFAP) around nuclei and in the apical process (scored as GFAP^+^ or GFAP^−^).

Within the EdU^+^BrdU^−^ cell subpopulation, we identified cells with five of the seven possible combinations of these features. The bulk of EdU^+^BrdU^−^ cells (more than 50%) had the GFP^+^GFAP^+^process^+^ phenotype, characteristic of *bona fide* RGLs (Figure 2B,D and Figure S2 and Table S1). A minor subpopulation (14.5%) of EdU^+^BrdU^−^ cells had a GFP^+^GFAP^−^process^−^ phenotype; these cells might have corresponded to pericytes, endothelial cells, oligodendrocyte progenitor cells and ANPs (Figure 2D and Figure S2 and Table S1). The EdU^+^BrdU^−^ cells with a GFP^−^GFAP^−^process^−^ phenotype (5.3%) did not display features of RGLs or ANPs; these cells of unidentified phenotype might potentially correspond to microglial and/or vasculature cells.

The remaining two phenotypes identified among the EdU^+^BrdU^−^ cells were GFP^+^GFAP^+^process^−^ cells (22.7%) and GFP^+^GFAP^−^process^+^ cells (4.7%) (Figure 2D and Figure S2 and Table S1). The larger cell fraction contained GFP-positive astrocyte-like cells, in which we were unable to unequivocally identify an apical process in the Z-stacks of optical sections; these cells might have included RGLs with a partially retracted or an obliquely positioned apical process. Thus, RGLs may represent up to ~75% of the EdU^+^BrdU^−^ cells. The smallest subpopulation was GFP^+^GFAP^−^process^+^ cells, which we designated as cells of unidentified phenotype (possibly including a subpopulation of oligodendrocyte progenitor cells). Together, these subpopulations corresponded to those expected from the detailed analysis of the SGZ cells [16,36,42].

Thus, our observations indicated that our double-labeling scheme enables identification of, and differentiation between, cells that have just entered their first cell cycle and those that have already undergone one or several rounds of division in the recent past. Furthermore, it allows for the identification of subclasses of dividing stem and progenitor cells in the adult hippocampus.

### 3.3. Cellular Mechanism Underlying AHN Enhancement by Memantine

We next applied our novel double-labeling scheme to determine the changes in stem cell division induced by memantine, a potent inducer of AHN. Memantine, a low-affinity non-competitive antagonist of the N-methyl-D-aspartate receptor, is used to ameliorate cognitive deficits in patients with Alzheimer’s disease [43,44]. Memantine has been shown to increase the number of dividing RGLs when administered to mice [45,46].

**Figure 3:**
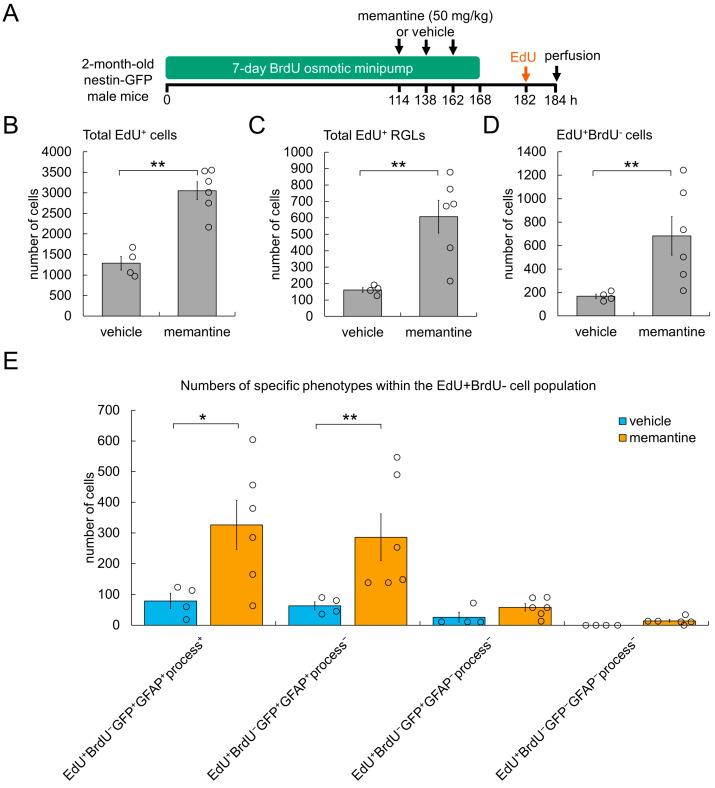
Memantine enhances proliferation in the DG of the adult brain via recruitment of quiescent RGL stem cells. (**A**) Scheme of the experiment, demonstrating delivery of two labels and treatment with memantine. Memantine significantly induces proliferation (**B**) and increases the numbers of proliferating RGLs (**C**) and de novo dividing cells (**D**) in the DG. (**E**) Phenotyping of EdU^+^BrdU^−^ cells, indicating that memantine significantly increases the numbers of de novo dividing *bona fide* (GFP^+^GFAP^+^process^+^) and presumptive (GFP^+^GFAP^+^process^−^) RGLs. Quantitative data are presented as mean ± SEM. Statistics: vehicle n = 4, memantine n = 6; Mann–Whitney test, * *p* < 0.05, ** *p* < 0.01.

In addition to BrdU delivery via implanted mini-osmotic pumps, we injected Nestin-GFP mice (2-month-old males) intraperitoneally with either memantine or vehicle (saline) three times with 24 h between injections, and then administered EdU ~20 h after the last injection of memantine, i.e., mice received the first memantine injection 3 days before the administration of EdU (Figure 3A). This regimen was chosen because an increase in RGL proliferation was observed on the third day after a single injection of memantine [42,43]. Because the detailed kinetics of the action of memantine on AHN is unknown, we injected mice with memantine three times to increase the probability of detecting its effect. Quantification of EdU^+^ cell nuclei in confocal images indicated that three daily injections of memantine, compared with the control, resulted in significantly greater cell proliferation in the SGZ (3051 ± 218 vs. 1286 ± 165 of EdU^+^ cell nuclei) (Figure 3B). Memantine treatment also increased the number of dividing RGLs (defined as cells of the EdU^+^GFP^+^GFAP^+^process^+^ phenotype), to 3.8-fold that of the control (607 ± 100 vs. 161 ± 14 of EdU^+^ RGLs) (Figure 3C). Next, in the same series of images, we identified EdU^+^BrdU^−^ nuclei (i.e., all de novo dividing cells). The number of EdU^+^BrdU^−^ nuclei was significantly (4.1-fold) higher in the

SGZ in mice treated with memantine than the control (683 ± 164 vs. 168 ± 19 of EdU^+^ BrdU^−^ cell nuclei) (Figure 3D). We next determined the changes in the number of EdU^+^BrdU^−^ cells in different cell subpopulations as described above (Figure 3E). We detected 4.1-fold more EdU^+^BrdU^−^ cells with the GFP^+^GFAP^+^process^+^ phenotype (i.e., de novo dividing true RGLs) in mice receiving memantine than control injections (326 ± 80 vs. 79 ± 25 cells). The number of presumptive EdU^+^BrdU^−^ RGLs with the GFP^+^GFAP^+^process^−^ phenotype was also significantly greater in mice treated with memantine than the control (286 ± 76 vs. 63 ± 14 cells). Other types of de novo dividing cells (EdU^+^BrdU^−^GFP^+^GFAP^−^process^−^ and EdU^+^BrdU^−^GFP^−^GFAP^−^process^−^ cells) represented minor subpopulations of the EdU^+^BrdU^−^ cells in both memantine- and vehicle-treated mice. The other three theoretically possible cell phenotypes were not identified in the brain slices of the animals in both groups.

Together, these results suggest that exposure to memantine increases the number of quiescent RGLs recruited into the cell cycle.

## 4. Discussion

Here, we present a novel double labeling method for the detection of de novo dividing stem cells in situ and, with this method, show that the potent AHN inducer memantine augments division of quiescent RGLs. Our novel labeling scheme advances stem cell research methods by providing an approach for assessing de novo dividing cells in situ. Our approach is based on a combination of cumulative and pulse labeling of dividing cells with two nucleotide analogues, and it enables discrimination between proliferating cells that have just exited the quiescent state and entered the cell cycle and those proliferating cells that have already undergone one or more rounds of division within the preceding period of time. Our scheme detects de novo dividing cells that enter their first S phase after a period of being outside of the cell cycle. Notably, our approach is not retrospective, and it allows for direct determination of the balance between quiescence and division and the changes in this balance.

Our technique could be exploited for the identification of de novo dividing cells within virtually any stem cell niche, including cancerous stem cell niches, in a large variety of organisms. Notably, this method also provides additional options for revealing the proliferative behavior of stem cells. For example, the period of the first label delivery could be readily extended by implanting a charged mini osmotic pump with prolonged release duration (up to 6 weeks, per the manufacturer’s specifications) to detect stem cells that have been quiescent for an extended period of time before their recruitment into the cell cycle. Furthermore, by extending the chase period after the second label pulse, fates could be traced for progenies originating from a cohort of stem cells that left the quiescent state and entered the cell cycle at a specific time point. Moreover, combining this approach with genetic tracing might improve the resolution of analysis of cellular mechanisms underlying tissue renewal and repair.

Our results based on this new technique provide new insights into the regulation of quiescence and division of neural stem cells in the adult brain. We found that most de novo dividing cells in the DG corresponded to RGLs, and that negligible numbers of dividing ANPs had not been dividing within the preceding 7 days. These results are in remarkable agreement with a model of stem cell maintenance [36,37] in which RGLs stem cells do not normally alternate between quiescence and proliferation, and ANPs are born from RGLs, thus implying that de novo dividing neurogenic cells in the DG should have the RGL phenotype.

We used our novel method to study the profound pro-neurogenic action of the anti-Alzheimer’s disease drug memantine. Our results suggest that, in the adult DG, memantine augments neurogenesis via increasing the recruitment of quiescent RGLs into the cell cycle. A potential implication of these results is that if the pool of hippocampal stem cells is not replenished, continued excessive recruitment of quiescent RGLs into the cell cycle may gradually deplete the pool, thus possibly leading to delayed detrimental outcomes. Indeed, overactivation of quiescent RGLs has been reported to elicit premature depletion of their pool, thereby considerably decreasing AHN in later stages of life [47,48,49]. Thus, our findings may be relevant for the practical evaluation of drug treatments and medical interventions in animal models in terms of their action on quiescent stem cells at different stages of life, as well the development of strategies for the prevention of treatment- or pathology-induced premature depletion of quiescent stem cells.

Our new method combines cumulative and pulse labeling with two thymidine analogues. Here, we used BrdU and EdU, which have been found, under certain conditions, to exhibit cytotoxic effects after their incorporation into the replicating DNA. These toxic effects vary depending on the dose, treatment duration, and time passed after treatment and may subsequently manifest as alterations in proliferative behavior, premature cell senescence, and aberrant differentiation [18,50,51]. However, we do not expect the cytotoxicity of BrdU and EdU to significantly affect the detection of de novo dividing stem cells in our experiments, for the following reasons. We delivered BrdU via mini osmotic pumps at a rate of 50 mg/kg per day. This delivery rate can be considered, at most, equivalent to a single intraperitoneal injection of BrdU at a dose of 50 mg/kg per day, which does not show an apparent detrimental effect on proliferating cells [51,52,53]. Moreover, we are focused on the population of EdU^+^BrdU^−^ cells (i.e., those that lack the BrdU label). Hence, any potential detrimental effects of BrdU should not affect the cell population of our interest. Furthermore, the short chase period (2 h) after the EdU administration is unlikely to lead to potential EdU-induced cytotoxic effects, which were found to manifest primarily after one round of cell division [53,54,55,56]. In experiments aimed to trace the fates of de novo dividing cells, when prolonged chase periods after the second label pulse may be necessary, other thymidine analogues such as (2S)-2-deoxy-2-fluoro-5-ethynyl uridine (F-ara-EdU) can potentially be used as the second label because F-ara-EdU was shown to be less toxic than EdU or BrdU [57].

Overall, our new method presents new opportunities for studying stem cells by enabling identification of de novo dividing stem cells in situ and tracing the fates of progenies originating from stem cells that exited quiescence and begin to divide at a specific time point. Our method enables assessment of how various factors, such as aging, pathology, drug treatment or medical intervention, may influence the balance between quiescence and division of stem cells.

## Data Availability

All data are contained within the article.

## Supplementary Materials

The following supporting information can be downloaded at: https://www.mdpi.com/article/10.3390/cells11244001/s1, Supplementary Figure S1: Detection of de novo dividing in the hippocampal dentate gyrus through double nucleotide analogue labeling; Supplementary Figure S2: Representative images of the identified phenotypes of EdU^+^BrdU^−^ cells. Supplementary Table S1: Percentages of specific cell phenotypes within the EdU^+^BrdU^−^ population.
