# Supplementary material for: Detection of De Novo Dividing Stem Cells In Situ through Double Nucleotide Analogue Labeling
Source: Cells. 2022 Dec 10;11(24):4001. doi: 10.3390/cells11244001 (PMC9777310; doi:10.3390/cells11244001)
Supplement: Supplementary file 1 [file cells-11-04001-s001.zip › cells-2010054-supplementary.pdf]

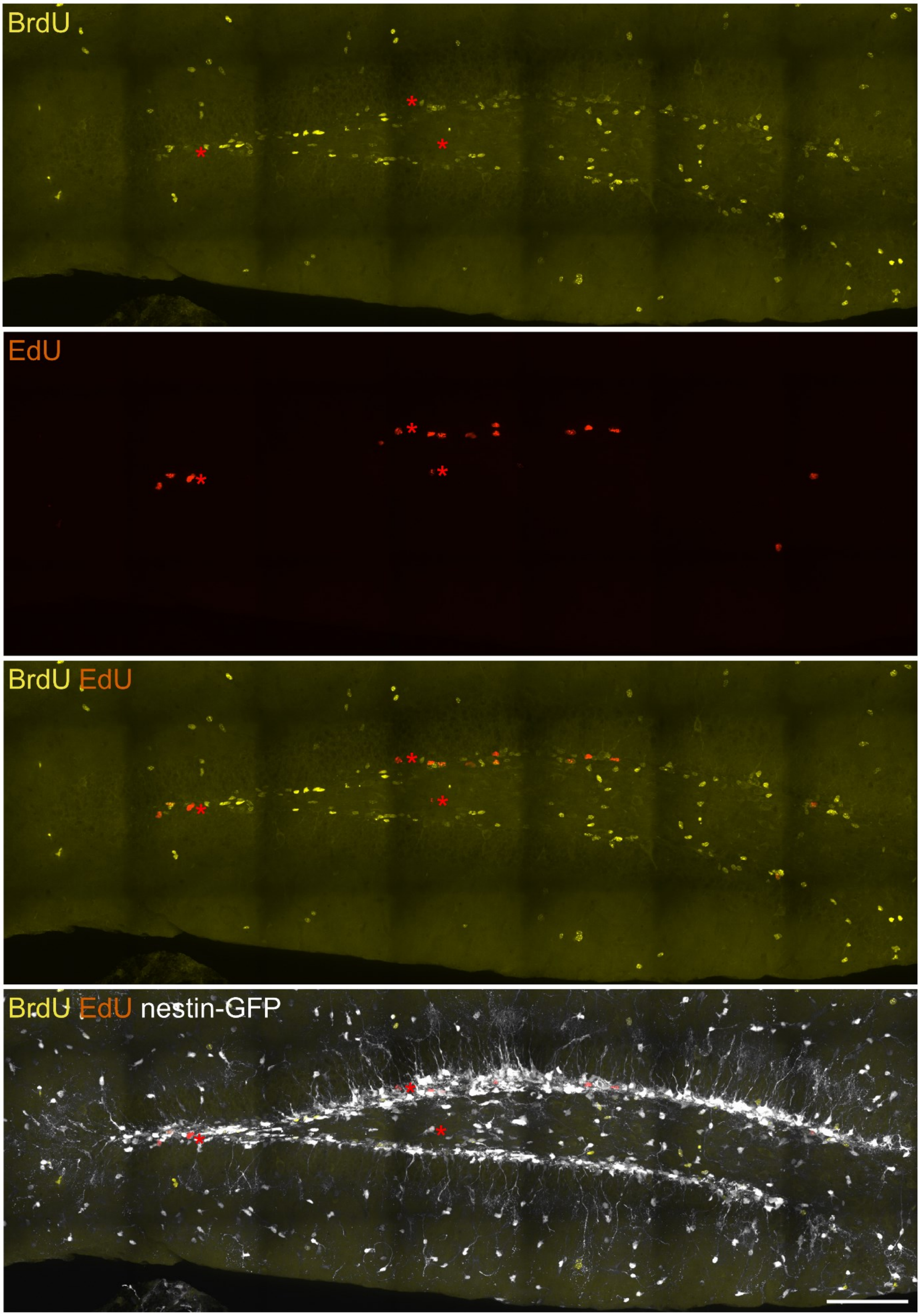

Supplementary Figure S1 (related to Figure 2B). Detection of *de novo* dividing in the hippocampal dentate gyrus through double nucleotide analogue labeling. The image is a maximal projection of a Z-stack captured with a confocal microscope. Red asterisks indicate $EdU^{+}BrdU^{-}$ nuclei. Scale bar: 100 µm.

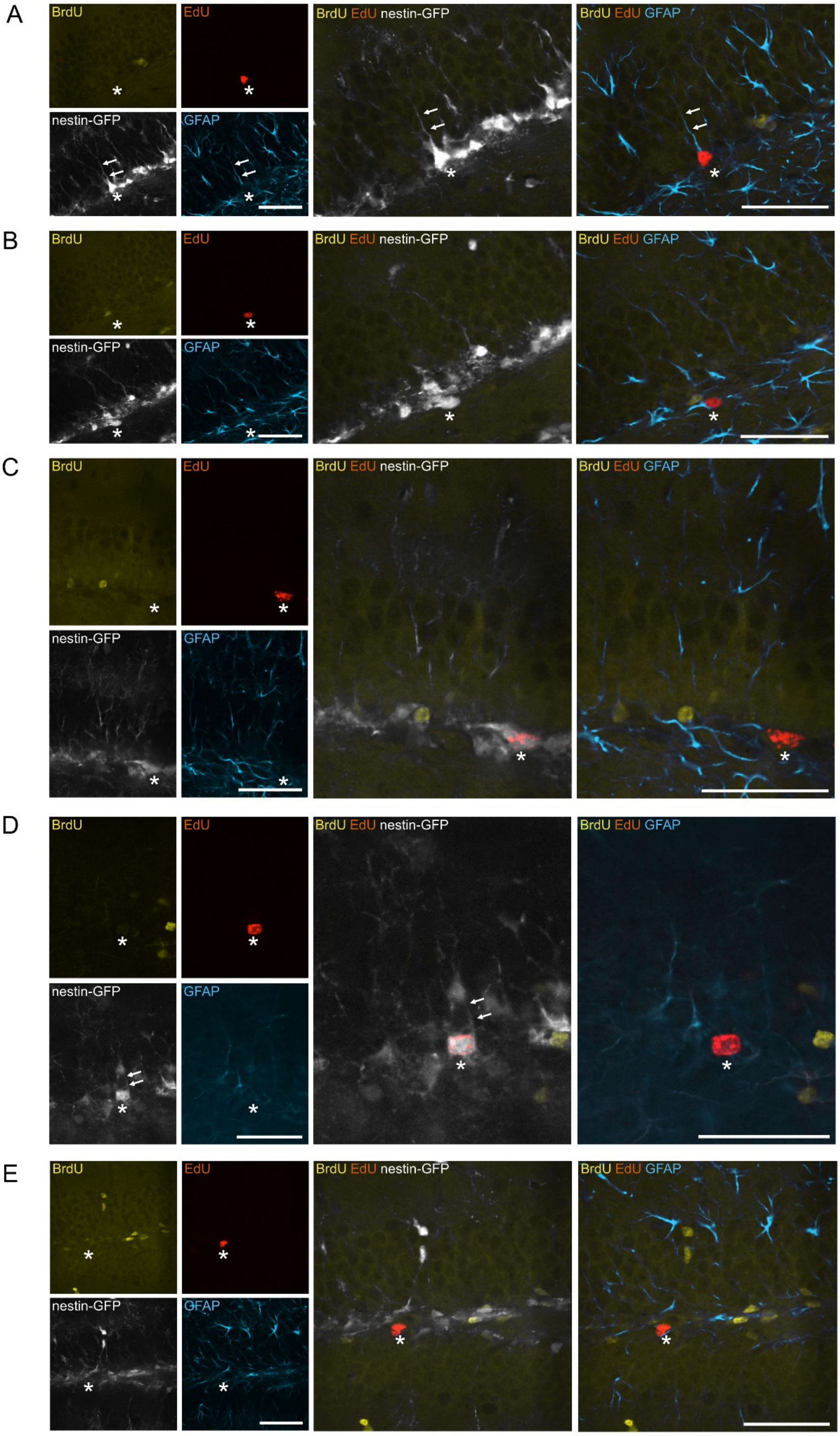

Supplementary Figure S2 (related to Figure 2D). Representative images of the identified phenotypes of EdU$^+$BrdU$^-$ cells. **A** GFP$^+$GFAP$^+$process$^+$ RGLs, **B** GFP$^+$GFAP$^+$process$^-$ presumptive RGLs, **C** GFP$^+$GFAP$^-$process$^-$, **D** GFP$^+$GFAP$^-$process$^+$, and **E** GFP$^-$GFAP$^-$process$^-$. Here, images are single optical section captured with a

confocal microscope. Asterisks indicate bodies of $EdU^{+}BrdU^{-}$ cells. Arrows indicate an apical process. Scale bars: 50 µm.

Supplementary Table S1 (related to Figure 2D). Percentages of specific cell phenotypes within the EdU^+BrdU^- population.

Phenotype	Percentages for individual mice (%)						mean (%)	SEM (%)
	1	2	3	4	5	6		
$EdU^+BrdU^-GFP^+GFAP^+process^+$ (RGLs)	57.2	41.7	50	72.6	25	70.5	52.8	7.3
$EdU^+BrdU^-GFP^+GFAP^+process^-$ (presumptive RGLs)	14.5	16.6	50	4.6	33.1	17.5	22.7	6.6
$EdU^+BrdU^-GFP^+GFAP^-process^-$	21.4	33.1	0	9.3	16.9	6	14.5	4.9
$EdU^+BrdU^-GFP^-GFAP^-process^-$	6.9	0	0	0	25	0	5.3	4.1
$EdU^+BrdU^-GFP^+GFAP^-process^+$	0	8.6	0	13.5	0	6	4.7	2.3
